# Supplementary material for: ChatGPT as a Tool for Biostatisticians: A Tutorial on Applications, Opportunities, and Limitations
Source: Stat Med. 2025 Oct 23;44(23-24):e70263. doi: 10.1002/sim.70263 (PMC12548020; doi:10.1002/sim.70263)
Supplement: Supplementary file 1 — Data S1. Supporting Information. [file SIM-44-0-s001.zip › Supplementary Materials Additional.zip!/3.5_Causal_Inferece/3.5_Causal_Chat6.pdf]

OpenAI

Combining statistical and causal thinking_6 - Biostatistics Expert Assistance

Please clear memory.

I've cleared the memory for this conversation. If you'd like me to remember anything again in the future—such as a project you're working on or your writing preferences—just let me know!

Take the role of an expert biostatistician for this Chat

Understood. I'll act as an expert biostatistician for this chat—ready to assist with study design, statistical analysis, interpretation, or methodological questions. Just let me know what you're working on or need help with.

I have data from a randomised clinical trial. I want to compare the efficacy of the experimental treatment with that of the control on overall survival in patients with non-small cell lung cancer. In this two-arm RCT, a considerable number of patients in the control arm switched to the experimental treatment after disease progression, while those in the experimental arm never switched.
My goal is to estimate the causal effect of sustained intake of the experimental treatment versus sustained intake of control on Overall survival, had the control group patients not been allowed to switch. Based on the information provided above, please define my target Estimand, following the guidance from the Estimand framework (ICH-E9 addendum)

Given your context—a two-arm randomised clinical trial in non-small cell lung cancer (NSCLC) with treatment switching in the control arm post-progression, and no switching in the experimental arm—your objective is to estimate the *causal effect* of sustained treatment with the experimental therapy versus sustained control, under a *hypothetical scenario where no switching occurs*. This aligns with the **hypothetical strategy** outlined in the **ICH E9 (R1) Estimand Framework**.

Below is a structured definition of your **target estimand**, using the ICH E9 (R1) framework's five attributes:

Target Estimand (Hypothetical Strategy)

1. **Treatment**:
   - *Treatment condition*: Sustained receipt of the **experimental treatment** as assigned at randomisation.

   - *Control condition*: Sustained receipt of the **control treatment** as assigned at randomisation, assuming patients did **not switch** to the experimental treatment after progression.
2. **Population**:
   - All randomised patients with **non-small cell lung cancer**, as defined by the trial's eligibility criteria (i.e., the **intention-to-treat (ITT) population**).
3. **Variable (Endpoint)**:
   - **Overall survival (OS)**, defined as the time from randomisation to death from any cause.
4. **Intercurrent Events and Strategy for Handling Them**:
   - The key intercurrent event is **treatment switching** from control to experimental after disease progression.
   - Strategy: **Hypothetical strategy** — estimate what OS would have been *had patients in the control arm not switched* to the experimental treatment.
5. **Summary Measure**:
   - A causal **hazard ratio** comparing OS under sustained experimental treatment versus sustained control (no switching), possibly estimated using **g-methods** such as inverse probability of censoring weighting (IPCW) or the g-formula.
   - Alternative summary measures could include **restricted mean survival time (RMST)** or **difference in survival probabilities** at a fixed time point (e.g., 12 months).

Optional — Plain-language description:

> *The estimand of interest is the treatment effect on overall survival in the full trial population, comparing a scenario in which all patients had received the experimental treatment continuously from randomisation, with a scenario in which all patients had remained on control treatment, assuming no switching to experimental therapy occurred after progression.*

If you'd like, I can help you articulate this definition for inclusion in a protocol, SAP, or publication. Let me know if you're considering IPCW, g-formula, or other methods for estimation—I can advise on assumptions and fit with your estimand.

The switching behaviour is affected by a set of different baseline and time-varying covariates. Here, I want to assume that the time-varying confounders that influence the switching behaviour and Overall survival are also themselves affected by prior treatment intake.
For e.g. ECOG score might not only affect whether patients should switch treatment or not, but its value might also be affected by prior treatment intake reflected by the switching behaviour.
In this case, the analytical problem becomes more complex. Can you explain?

Yes, the analytical problem indeed becomes more complex—and you're correctly identifying a classic **time-varying confounding with treatment-confounder feedback** scenario. This arises frequently in oncology trials with treatment switching, and it has important implications for **causal estimation**.

Let's walk through the key concepts and why standard methods fail in this setting:

🔁 Time-Varying Confounding Affected by Prior Treatment

You've described a setting where:

- Certain **time-varying covariates** (e.g., ECOG score, tumour burden, progression status) affect both:
  - the **decision to switch** from control to experimental treatment, and
  - the **survival outcome** (OS).
- These **covariates are themselves influenced** by **prior treatment history** (e.g., control vs. experimental, or the act of switching).

This feedback loop creates what's known as:

> **Treatment-confounder feedback**

❌ Why Standard Methods Fail

1. **Time-varying Cox models** or **standard regression with time-dependent covariates** typically **adjust for these covariates directly**, but doing so **blocks part of the causal pathway** from treatment to outcome, leading to:
   - **Bias due to collider stratification** (conditioning on a variable affected by prior treatment and affecting future outcomes), and
   - **Inconsistent effect estimates** for the causal effect of interest.
2. **Intention-to-treat (ITT) analysis** ignores switching and underestimates the treatment effect.
3. **Per-protocol or naïve censoring** approaches also suffer from **selection bias** if switching is not random.

✅ Appropriate Analytical Solutions

To validly estimate the **causal effect of sustained treatment strategies** in the presence of time-varying confounding affected by prior treatment, you need to use **g-methods** developed in the causal inference framework:

1. Inverse Probability of Censoring Weighting (IPCW)

- Treats switching as a form of censoring.
- Models the probability of remaining on assigned treatment over time, conditional on past covariates.
- Applies weights to create a pseudo-population in which treatment switching is independent of observed covariates.
- Requires:
  - No unmeasured confounding.
  - Correct specification of the censoring model.

- Positivity (non-zero probability of remaining uncensored at each time point).

2. G-formula (Parametric g-computation)

- Explicitly models the joint distribution of time-varying covariates, treatment, and outcome.
- Simulates the outcome under hypothetical sustained treatment strategies.
- Requires full specification of the longitudinal data-generating process.

3. G-estimation of Structural Nested Models (SNMs)

- Less common in practice but powerful.
- Suitable when treatment is given at multiple time points with time-varying confounding.

🧠 Conceptual Summary

Because switching is **non-random and driven by patient characteristics** (some of which evolve over time and are affected by treatment), simple comparisons or regression adjustments are **inadequate** and often misleading.

Instead, you need **causal inference methods** that:

- Respect the **temporal order** of events,
- Account for **time-varying confounding**, and
- Preserve the **causal interpretation** of your estimand (e.g., OS under sustained control vs. sustained experimental).

Would you like help framing this explanation in a manuscript, presentation, or protocol language? I can also help specify the models you might use for IPCW or g-formula based on your covariate set.

Depict our research problem in a DAG

Here is a **Directed Acyclic Graph (DAG)** that visually represents your research problem: a randomised controlled trial in NSCLC with **treatment switching in the control arm after progression**, **no switching in the experimental arm**, and **time-varying confounding affected by prior treatment**.

📌 Variables:

- **A_0**: Randomised treatment assignment (0 = control, 1 = experimental)
- **L_0**: Baseline confounders (e.g., age, ECOG, CNS involvement)
- **L_1**: Time-varying covariates (e.g., updated ECOG, tumour burden, progression)
- **A_1**: Post-baseline treatment (e.g., switch to experimental after progression in control arm)
- **Y**: Outcome (Overall Survival)

DAG:

```css
L₀ → A₀ ──────────────→ A₁ ──────────→ Y
│     │                  ▲              ▲
▼     ▼                  │              │
L₁ ←──┘                  └──────────────┘
▲
└──────── A₀ ────────────┘
```

🔍 Explanation of Arrows:

- **L_0 → A_0**: Baseline variables may be associated with randomisation strata, but due to randomisation, this is typically blocked (here shown for completeness).
- **A_0 → L_1**: Initial treatment assignment affects subsequent disease status and patient condition (e.g., ECOG score), creating **treatment-confounder feedback**.
- **L_1 → A_1**: Time-varying covariates influence switching decisions (control patients switch if they progress or deteriorate).
- **A_1 → Y**: Post-baseline treatment affects survival.
- **L_1 → Y**: Current disease state and patient condition impact survival.
- **A_0 → A_1**: Only control patients are eligible to switch (experimental patients do not switch).
- **A_0 → Y**: Initial treatment may have a direct effect on OS, even without switching.
- **L_0 → L_1** and **L_0 → Y**: Baseline factors affect disease trajectory and survival.

⚠️ Key Structural Feature:

- **Time-varying confounding affected by prior treatment**: L_1 is a **confounder** of A_1 → Y, but it is also **affected by A_0**, creating **treatment-confounder feedback**. This violates the assumptions of standard regression and Cox models, which is why **g-methods** are required.

Would you like this DAG in a publishable format (e.g., TikZ, DAGitty code, or PNG)? Or shall I add annotations to illustrate estimation strategies like IPCW or g-formula?

I would like to apply the inverse probability of censoring weighting (IPCW) approach to address the bias introduced by switching following the methodology discussed in the following paper: Robins JM, Finkelstein DM. Correcting for noncompliance and dependent censoring in an AIDS clinical trial with inverse probability of censoring weighted (IPCW) log-rank tests. Biometrics. 2000;56(3):779–88.
Briefly summarise the key points from this paper

Certainly. The 2000 paper by **Robins and Finkelstein** is a foundational reference for the **Inverse Probability of Censoring Weighting (IPCW)** approach to handle **dependent censoring and**

noncompliance in clinical trials. Here is a brief but comprehensive summary of the key contributions and ideas from the paper:

📄 Key Points from Robins & Finkelstein (2000) – IPCW in AIDS Trial Context

🎯 Objective

- To address **treatment noncompliance** and **dependent censoring** (censoring that depends on patient characteristics) in randomised clinical trials.
- To propose a **causal method**—IPCW—that enables valid inference under a **hypothetical strategy** (e.g., if all patients had remained on their assigned treatment).

🔍 Problem Setting

- In AIDS trials, patients assigned to the control arm sometimes **crossed over** to experimental treatment due to disease progression or physician judgement.
- Switching (or noncompliance) may **depend on time-varying prognostic factors**, which also influence survival, creating **informative (dependent) censoring** when using per-protocol or naïve approaches.

🧠 Methodological Innovation: IPCW Log-Rank Test

1. **Key Idea**:
   Artificially **censor** patients at the time they **switch from their assigned treatment** (i.e., deviate from protocol), and then **reweight** the remaining individuals to correct for potential **selection bias** due to non-random censoring.
2. **Inverse Probability Weights**:
   - Weights are calculated as the **inverse of the probability** of remaining uncensored (i.e., compliant) up to time *t*, **conditional on covariate and treatment history**.
   - These weights create a **pseudo-population** where censoring is independent of prognosis, thus removing bias.
3. **Estimation**:
   - The IPCW method is used to construct **weighted versions of standard survival analyses**, including the **log-rank test** and **Kaplan-Meier curves**.
   - The survival function and log-rank test statistics are computed using these weights.

📐 Assumptions

To yield **unbiased causal estimates**, the method relies on:

1. **No unmeasured confounding**:
   All predictors of both switching and the outcome must be **measured and correctly included** in the weighting model.
2. **Correct model specification**:
   The model used to estimate censoring probabilities (often a pooled logistic regression) must be correctly specified.
3. **Positivity**:
   There must be a **non-zero probability** of remaining uncensored for all relevant covariate histories.

📈 Benefits and Use Cases

- Enables **causal interpretation** under a **hypothetical strategy**: what would have happened had patients remained on their assigned treatment.
- Particularly useful in **oncology and infectious disease trials** where treatment switching is common.
- Robust alternative to naïve per-protocol or as-treated analyses.

⚠️ Limitations and Challenges

- Requires **longitudinal data** on time-varying covariates.
- Sensitive to **model misspecification** and **extreme weights** (may require truncation or stabilisation).
- Does not address **unmeasured confounding**; external data or sensitivity analyses may be needed.

✅ In Summary:

Robins & Finkelstein (2000) introduced a rigorous, model-based approach for correcting selection bias due to informative censoring using IPCW. Their method allows for causal survival comparisons under a sustained treatment strategy, especially in settings where switching is driven by evolving prognostic indicators.

Let me know if you'd like help applying this method to your trial data—including specifying the censoring model, creating weights, checking positivity, or implementing the weighted survival analysis.

Could you elaborate more on the research question Robins and Finkelstein were trying to answer in their paper, mentioning the comparator arms, possible intercurrent events, outcome definition and population under study.

Certainly. Here's a more detailed elaboration of the **research question** and setting addressed by **Robins and Finkelstein (2000)** in their IPCW methodology paper, framed using the language of modern clinical trial design (including concepts like comparator arms, intercurrent events, outcome, and target population):

📄 Research Context from Robins & Finkelstein (2000)

🎯 Primary Research Question

> *What is the causal effect of assignment to early versus deferred zidovudine (AZT) treatment on overall survival in HIV-infected individuals with a CD4 count below 200, had all patients adhered to their assigned treatment strategy?*

In modern terms, this is a **hypothetical estimand**: what would the survival comparison look like **if no one deviated from their assigned treatment strategy**, despite actual noncompliance in the trial.

🧪 Comparator Arms

- **Early AZT (Experimental Arm)**: Immediate initiation of zidovudine treatment after randomisation.
- **Deferred AZT (Control Arm)**: Initiation of zidovudine treatment only after progression to AIDS or some other predefined criterion.

Both arms were assigned at **baseline via randomisation**.

🔀 Intercurrent Event

- **Noncompliance / Treatment Switching**:
  - Some patients in the **deferred treatment arm** initiated AZT **earlier than assigned**, often due to rapid health deterioration or physician judgement.
  - This **violated the protocol** and represented a **treatment switch**, which was **not random**—it was influenced by **time-varying prognostic factors** (e.g., declining CD4 count, worsening symptoms).

This creates **dependent (informative) censoring** if patients are censored at switch, as those who switch early are likely different in prognosis.

⚰️ Outcome

- **Overall Survival (OS)**:
  - Time from randomisation to **death from any cause**.
  - A hard clinical endpoint, not subject to measurement error, and considered central in HIV/AIDS clinical trials.

👥 Population

- The **intention-to-treat (ITT) population**:
  - All individuals **randomised** to either early or deferred treatment, regardless of subsequent adherence.

- The analysis seeks to recover what the **per-protocol survival comparison** would have looked like in the **entire randomised population**, under **full adherence**.

🧠 Analytical Goal

- Estimate the **causal effect of treatment assignment on survival**, **under the hypothetical scenario** in which **everyone adhered** to the treatment protocol (no early AZT initiation in the deferred group).
- Account for the **informative nature** of switching, which depends on evolving patient status (i.e., time-varying confounders).

📌 Summary in Modern ICH E9(R1) Terms:

Estimand Attribute	Specification
**Treatment**	Early vs. deferred AZT initiation
**Population**	All randomised patients with CD4 < 200
**Variable (Endpoint)**	Time to death from any cause (OS)
**Intercurrent Event**	Early initiation of AZT in deferred arm (noncompliance)
**Strategy**	**Hypothetical**: estimate survival as if all adhered to assigned strategy
**Summary Measure**	Survival curves and log-rank test using IPCW

Let me know if you'd like to see how this parallels your lung cancer trial, or how to translate this logic into a statistical analysis plan or estimand table for your own study.

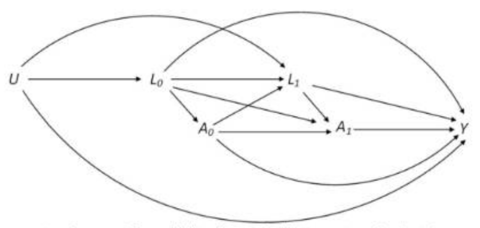

Fig. 1 Graphical representations of our research focus and the bias of naive methods for time-varying confounding in the presence of treatment-confounder feedback: This Directed acyclic graph (DAG) represents the post-progression period for patients in the control arm. Following disease progression, the decision as to whether or not a patient in the control group continues to take its treatment (A_0) or (A_1) depends on predictive factors measured at or before the time of disease progression (L_0). Again, we assume that treatment intake (A_0) affects outcome (Y) and influences a set of TVCs at a later time-point (L_1). These covariates are also assumed to impact the clinician's decision as to whether or not a patient should subsequently change treatments (A_1) introducing time-varying confounding and treatment-confounder feedback through L_1

Attached, I provide you with a directed acyclic graph (DAG) that represents our research problem.
To simplify this DAG and take into consideration this deterministic information provided below, I restrict my DAG to the post-progression period for patients in the control arm only and consider two follow-up time points, 1 & 2.
Here A(k) is deterministically 0 if A(k-1)=0 AND L(k-1) (reflecting progression status)=0 (no progression).
Following disease progression, the decision as to whether or not a patient in the control group continues to take their treatment (A0 or A1) depends on predictive factors measured at or before the time of disease progression (L0).
Again, we assume that treatment intake (A0) affects outcome (Y) and influences a set of TVCS at a later time-point (L1).

These covariates are also assumed to impact the clinician’s decision as to whether or not a patient should subsequently change treatments (A1), introducing time-varying confounding and treatment confounder feedback through L1.
I will emphasise again that in our case scenario, patients in the control group do not switch unless they progress, and all patients in the experimental group never switch.
I still want to estimate in my outcome model the causal effects of sustained treatment interventions (always taking control versus always taking experimental) under a hypothetical strategy where control patients do not switch treatment.
I want you to use this DAG as a basis for performing the appropriate IPCW analyses and provide me with the analysis R-script.
You may assume that the dataset is in long format, with variables: id, time (in months and increases with increments of 1), death indicator, switching indicator, baseline covariates
repeated over time points, time-varying covariates at each time pt.

DAG representing post-progression period for control arm

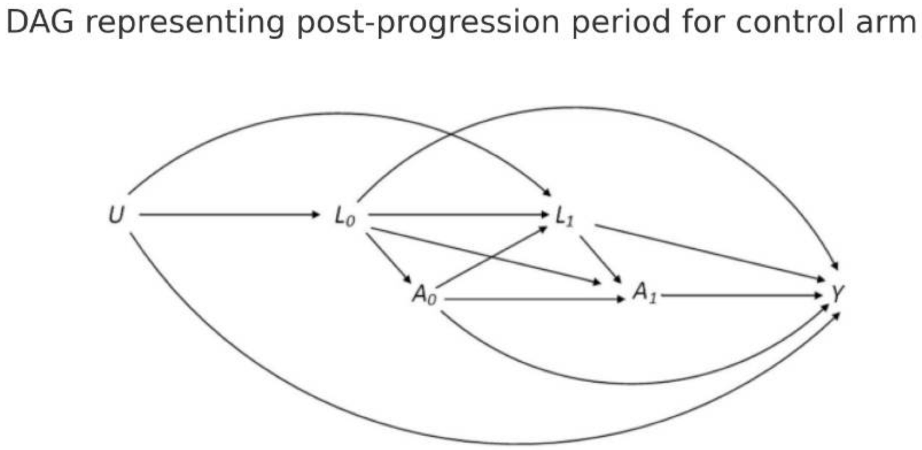

Fig. 1 Graphical representations of our research focus and the bias of naive methods for time-varying confounding in the presence of treatment-confounder feedback: This Directed acyclic graph (DAG) represents the post-progression period for patients in the control arm. Following disease progression, the decision as to whether or not a patient in the control group continues to take its treatment (A_0) or (A_1) depends on predictive factors measured at or before the time of disease progression (L_0). Again, we assume that treatment intake (A_0) affects outcome (Y) and influences a set of TVCs at a later time-point (L_1). These covariates are also assumed to impact the clinician's decision as to whether or not a patient should subsequently change treatments (A_1) introducing time-varying confounding and treatment-confounder feedback through L_1

Thank you for providing the DAG and description. Based on your setup, here is a tailored **R script** to implement **IPCW for treatment switching**, using your long-format data and aligning with the assumptions from your DAG and Robins & Finkelstein (2000).

📦 Assumptions

- Dataset is in **long format**: one row per patient per time interval.
- Key variables:
  - `id`: patient identifier
  - `time`: follow-up time in months
  - `death`: event indicator (1 = death, 0 = censored)
  - `switch`: treatment switching indicator (1 = switched, 0 = not switched)
  - `A`: treatment received at time `t` (0 = control, 1 = experimental)
  - `L0_*`: baseline covariates (time-invariant)

- `L1_*`: time-varying covariates (e.g., ECOG, progression status)

📜 IPCW Analysis Script in R

```r
# Required packages
library(survival)
library(ipw)
library(dplyr)
library(splines)

# Load your long-format dataset
# df <- read.csv("your_data.csv") # assume data is already loaded as 'df'

# Step 1: Define time of switch
df <- df %>%
  group_by(id) %>%
  mutate(switched = max(switch),
         time_of_switch = ifelse(switched == 1, min(time[switch == 1]), NA))

# Step 2: Define censoring indicator
df <- df %>%
  mutate(
    censor = ifelse(A == 0 & switch == 1, 1, 0),  # artificially censor at switch
    event = ifelse(censor == 1, 0, death)
  )

# Step 3: Fit model for probability of not being censored
# Create a pooled logistic regression model for censoring weights
df <- df %>%
  mutate(
    time_factor = as.factor(time),  # discrete time points
    uncensored = 1 - censor
  )

# You can include all relevant baseline and time-varying covariates
censor_model <- glm(
  uncensored ~ time + L0_age + L0_ecog + L1_ecog + L1_prog_status + ns(time, 3),
  data = df,
  family = binomial()
)

# Step 4: Compute predicted probabilities and weights
df$prob_uncensored <- predict(censor_model, type = "response")

# Stabilised weights (optional): use marginal model for numerator
marginal_model <- glm(
  uncensored ~ ns(time, 3),
  data = df,
  family = binomial()
)
df$prob_marginal <- predict(marginal_model, type = "response")

df <- df %>%
  group_by(id) %>%
  mutate(
    sw = cumprod(prob_marginal) / cumprod(prob_uncensored),  # stabilised weights
    ipcw_weight = ifelse(is.na(sw), 1, sw)
  )

# Step 5: Fit weighted survival model (e.g., weighted Cox model)
cox_ipcw <- coxph(
  Surv(time, event) ~ A,
  data = df,
  weights = ipcw_weight,
  robust = TRUE  # robust variance
)
```

```
summary(cox_ipcw)
```

📌 Notes

- **Modeling the censoring**: The quality of IPCW depends on the correct specification of the censoring model. Include relevant **baseline and time-varying covariates** (those affecting both switching and survival).
- **Stabilised weights**: Help reduce variance and mitigate the impact of extreme weights.
- **Time scale**: Assumes discrete time steps (monthly). If you use continuous time, adjustments are needed.
- **Survival model**: Could be a Cox model or weighted Kaplan-Meier depending on your estimand.

Let me know if you'd like help:

- Implementing **weight truncation** to control variance.
- Including **baseline strata or randomisation factors**.
- Adapting this to a **g-formula** or **pseudovalue regression** instead.

I'm also happy to help test or refine this with sample data.
